# Depression in Children and Adolescents on the Qinghai-Tibet Plateau: Associations with Resilience and Prosocial Behavior

**DOI:** 10.3390/ijerph18020440

**Published:** 2021-01-08

**Authors:** Buzohre Eli, Yueyue Zhou, Yiming Liang, Jin Cheng, Jiazhou Wang, Changbing Huang, Xi Xuan, Zhengkui Liu

**Affiliations:** 1 CAS Key Laboratory of Mental Health, Institute of Psychology, Chinese Academy of Sciences, Beijing 100101, China; ail@psych.ac.cn (B.E.); zhouyueyue@psych.ac.cn (Y.Z.); liangym@psych.ac.cn (Y.L.); wangjz@psych.ac.cn (J.W.); 2 Department of Psychology, University of Chinese Academy of Sciences, Beijing 100049, China; 3 School of Psychology, Beijing Sport University, Beijing 100084, China; chengjin2020@163.com; 4 CAS Key Laboratory of Behavioral Science, Institute of Psychology, Chinese Academy of Sciences, Beijing 100101, China; huangcb@psych.ac.cn; 5 Department of Law and Politics, Nankai University Binhai College, Tianjin 300270, China; xuanxi123456@126.com

**Keywords:** depression, resilience, prosocial behavior, children and adolescents, Qinghai-Tibet Plateau

## Abstract

Depression in children and adolescents has become a serious public health problem worldwide. The objectives of this study were twofold: first, to investigate the status of depression among children and adolescents on the Qinghai-Tibet Plateau, the highest plateau in the world, with an average altitude of more than 4200 m (13,776 feet), and second, to examine the associations among prosocial behavior, resilience, and depression. A cross-sectional study was conducted among children and adolescents from Yushu Prefecture on the Qinghai-Tibet Plateau. A total of 11,160 participants aged 10–17 years (*M*_age_ = 14.34 years, *SD* = 1.77; 51.4% girls) were included. Self-reported depression, resilience, and prosocial behavior were assessed. The prevalence of depression was 29.2% in the current study. Higher levels of prosocial behavior were significantly associated with lower levels of depression (*β* = −0.25, *p* < 0.001). Furthermore, resilience significantly moderated the relationship between prosocial behavior and depression (*β* = −0.08, *p* < 0.001); that is, resilience enhanced the protective role of prosocial behavior in depression. These findings indicate that resilience may play an important role in the associations between prosocial behavior and depression, which suggests that improving resilience is essential for the prevention and intervention of depression among children and adolescents on the Qinghai-Tibet Plateau.

## 1. Introduction

Depression is common among children and adolescents worldwide and is the third leading cause of disability in adolescents globally [1]. A meta-analysis of 52 studies in over 20 countries estimated that the prevalence of depression among children and adolescents was 6–41% [2]. A considerable number of Chinese children and adolescents (19.8–24.3%) display symptoms of depression based on meta-analysis results [3,4]. In addition, previous studies have suggested that individuals at high altitudes have a higher prevalence of depression [5,6], as the unique geographical environment and living conditions are associated with high altitudes. Specifically, for individuals residing at moderate to high altitudes, the resultant hypoxemia and changes in atmospheric pressure, pharmacokinetics, and metabolism could reduce brain levels of serotonin [7], which affect an individual’s mood and emotion regulation [8]. Moreover, mental health services in high-altitude areas remain scarce due to structural issues, such as inconvenient transportation and underdeveloped economies [9]. The harsh natural environment and poor environmental and living conditions at high altitudes may also exacerbate the vulnerability of children and adolescents to depression due to their low adaptability and coping skills in this developmental period [10]. Therefore, depression among children and adolescents at high altitudes is particularly worthy of attention.

The Yushu Tibetan Autonomous Prefecture is located in the eastern hinterland of the Qinghai-Tibet Plateau, the highest plateau in the world, with an average altitude of more than 4200 m (13,776 feet). Due to the rugged geographical environment of the plateau and the relatively poor economic conditions, analyses of the mental health status of those in the Yushu Prefecture have not been optimistic [11,12]. In one study, 28.6% of Tibetan adults in the Yushu Prefecture on the Qinghai-Tibet Plateau displayed symptoms of depression [13]. To date, no study has reported the prevalence of depression and associated factors among children and adolescents on the Qinghai-Tibet Plateau.

Prosocial behavior may influence the development of depression. Prosocial behaviors are defined as voluntary behaviors intended to benefit others, including sharing, cooperating, helping, and caring [14]. Prosocial behaviors are central to the formation and maintenance of healthy interpersonal relationships and to integrate into society [15]. The arousal cost-reward model explains prosocial behavior from a cognitive perspective and believes that people will make cognitive decisions before showing prosocial behavior [16]. It also suggests that people engage in prosocial behavior to maximize rewards and minimize losses. The rewards for showing prosocial behavior may be to improve the status, bring honor, and improve mood [17]. From the perspective of the arousal cost-reward model, we, therefore, hypothesized that prosocial behavior might be beneficial, maximize rewards and thereby improve positive emotion, including reducing depression.

Moreover, previous empirical studies have found that prosocial behavior is positively related to positive affection [18] and protects against negative feelings [19], depression and anxiety disorder [20,21]. Studies with children and adolescents have also shown that prosocial behavior is negatively related to internalization problems, including depression and loneliness [22,23]. Individuals with low prosocial behavior face more interpersonal problems and stress [15], which may increase the risk of depression. A study reported that Tibetan residents of the Qinghai-Tibet Plateau are enthusiastic and help others [24], suggesting the possibility of higher prosocial behavior in this region and a more obvious protective effect of prosocial behavior on depression.

Factors that moderate the effects of protective factors for depression are also of interest. One factor that may enhance the association between prosocial behavior and depression is resilience. Resilience can be defined as protective or positive processes that reduce maladaptive outcomes under conditions of risk, limit bad behaviors, and promote good adjustment and development [25]. Individuals with high levels of resilience have been shown to exhibit fewer emotional and behavioral problems [26]. Resilience is also characterized by a high level of positive emotion [27]. According to the broaden-and-build theory, positive emotions may broaden an individual’s thought-action repertoire, expanding the range of cognitions and behaviors that come to mind [28]. These broadened mindsets, in turn, help individuals build lasting personal resources, including physical, intellectual, psychological, and social resources [28]. That is, resilience is not an outcome in itself, but instead a positive resource that individuals can utilize when challenged to accomplish positive outcomes [29]. A resilient individual may have sufficient internal resources (e.g., optimism, self-reliance, and self-efficacy) and external resources (e.g., social support) to avoid the negative impact of risk [30]. Overall, resilience is an important personal resource for helping individuals achieve good adjustment and development and has been found to improve mental health [31].

Furthermore, resilience has been suggested to be a possible protective factor against depression. Resilience may protect against depression not only by lessening or counteracting the negative impact of risk factors for depression [32,33] but also by enhancing the effects of favorable factors [34]. For instance, high resilience has been found to buffer the negative effects of childhood abuse and ostracism on depression among adolescents [33,35]. Resilience also enhances the protective roles of social support in depression among migrant children [34]. Notably, researchers have not yet explored the moderating role of resilience in the relationship between prosocial behavior and depression.

Based on the literature, the current study had two main aims. First, we investigated the status of depression in a large sample of children and adolescents on the Qinghai-Tibet Plateau. Second, we explored the associations between prosocial behavior, resilience, and depression. Consistent with previous studies, we hypothesized that children and adolescents living at high altitudes might be at elevated risk for depression. In addition, we hypothesized that higher levels of prosocial behavior would be associated with lower levels of depression. Finally, we expected that resilience might function as a moderator of the relationship between prosocial behavior and depression. Specifically, we anticipated a stronger effect of prosocial behavior on depression in individuals with high resilience than in those with low resilience.

## 2. Methods

### 2.1. Participants

**Figure 1:**
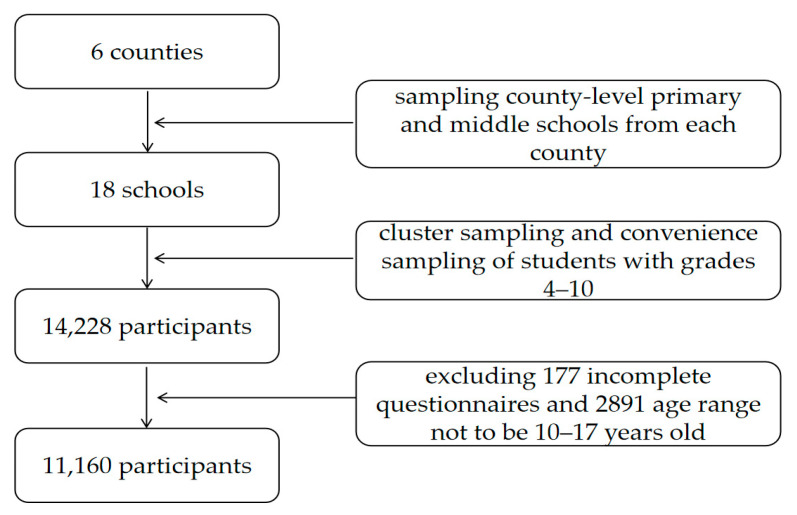
Flow chart of sampling.

The study was conducted in the Yushu Tibetan Autonomous Prefecture on the Qinghai-Tibet Plateau, China. There are six county-level administrative regions in the Yushu Prefecture (e.g., Nangqian County, Qumalai County, Zaduo County, Zhiduo County, Yushu County, and Chengduo County). Eighteen county-level schools (six primary schools with grades 4–6 and twelve middle schools with grades 7–10) in six county-level administrative regions in the Yushu Prefecture were selected for investigation. In this study, a total of 14,228 participants were investigated by using combined cluster sampling and convenience sampling. Of these, 177 (1.2%) participants were excluded due to incomplete or inaccurate responses. The inclusion criteria were age 10 to 17 years old in the current study. Therefore, 2891 (20.3%) were excluded due to age range not to be 10–17 years old. The final study sample consisted of 11,160 (78.5%) children and adolescents. The flow chart of sampling is shown in Figure 1, and the sociodemographic characteristics of the final study sample are presented in Table 1.

### 2.2. Measures

Depression was measured by the Center for Epidemiological Studies Depression Scale (CES-D-10) [36]. The CES-D-10 is a short form that consists of 10 items from the original 20-item Likert scale questionnaire assessing depressive symptoms in the past week. The options for each item range from 0 to 3 [0 = Rarely or none of the time (less than 1 day); 1 = Some or a little of the time (1–2 days); 2 = Occasionally or a moderate amount of time (3–4 days); and 3 = Most or all of the time (5–7 days)]. A higher score indicated more depressive symptoms, with a cutoff score of 10 or higher indicating the presence of significant depression [36]. The CES-D-10 has been shown to be an acceptable self-reported tool to screen depression in children and adolescents in community and nonclinical settings [37,38]. The Chinese version of the CES-D-10 also has been validated and widely used in Chinese children and adolescents [39]. In the present study, Cronbach’s alpha value for this measure was 0.90.

Resilience was measured by the short version of the Connor–Davidson resilience scale (CD-RISC) [40], which was originally developed by Connor and Davidson [25]. The short version of the CD-RISC is a 10-item scale that measures the ability to cope with stress and adversity. Items were rated on a 5-point Likert scale ranging from 1 (*never*) to 5 (*always*). Higher scores indicated high levels of resilience. The Chinese version of the CD-RISC has been validated and widely used in Chinese populations [41]. In the present study, Cronbach’s alpha value for this measure was 0.96.

Prosocial behavior was measured by the prosocial scale of the strengths and difficulties questionnaire (SDQ) [42]. The prosocial scale assesses adolescents’ prosocial behavior in the last 6 months based on responses to 5 items, which describe the positive behavior of children when they deal with others (e.g., showing consideration for others’ feelings). Items were rated on a 3-point Likert scale ranging from 1 (not true) to 3 (certainly true). Higher scores reflected positive prosocial behavior [43]. The Chinese version of the SDQ has shown good reliability and validity in a Chinese adolescent sample [44]. In the present study, Cronbach’s alpha value for this measure was 0.82.

Demographic information included gender (0 = girls, 1 = boys), age, and education level of the fathers and mothers (0 = never went to school, 1 = primary school, 2 = junior high school, and 3 = high school and above).

### 2.3. Procedure

This large-scale cross-sectional survey was conducted by the Institute of Psychology of the Chinese Academy of Sciences and the National Health and Family Planning Commission’s Institute of Science and Technology. The main aim was to understand the mental health status of children and adolescents in the Yushu Tibetan Autonomous Prefecture on the Qinghai-Tibet Plateau, China. Data collection was completed from September to November 2016. Written informed consent was obtained from all participants and their guardians (e.g., parents, immediate relatives), as well as school administrators, before data collection. All the research assistants received standardized and strict training to supervise the investigation. They introduced the survey purposes and provided prompt instruction to participants during the tests. After a complete description of the survey, the participants were assured that their responses would be kept completely confidential. The trained research assistants administered the questionnaires using the same procedures. A paper-pencil questionnaire was completed during class under the supervision of the researcher. All participants completed the questionnaires voluntarily. After the investigation, all the participants were entitled to a free medical examination. The survey protocol was approved by the ethics review committee of the Institute of Psychology, Chinese Academy of Sciences (project identification code: H16014).

### 2.4. Statistical Analyses

First, Little’s missing completely at random (MCAR) test [45] was used to assess the pattern of missing data. The results revealed that data were missing at random (*χ*^2^_1778_ = 3409.55, *p* = 0.07). Additionally, for the missing data, the expectation maximization (EM) algorithm in SPSS was imputed. Second, continuous variables are described using the mean and standard deviation (*SD*), and categorical variables are described using frequencies with percentages. Third, Pearson’s correlations were calculated between depression, resilience, prosocial behavior, age, gender, and parents’ education level. The above analyses were conducted in SPSS 21.0 (IBM, Armonk, NY, USA). Finally, the moderating roles of resilience were analyzed in the PROCESS plug-in (version 3.0 by Hayes, A.F.) [46]. The total score from the depression assessment served as a dependent variable. Prosocial behavior was an independent variable, and resilience was entered as a moderator. Simple regression lines were plotted to illuminate the moderation effect. Demographic variables that could be significantly related to depression were covariates [47]. All the continuous variables used in the PROCESS were conducted using standardized z-scores due to the differences in the scaling of the measures. Standardizing makes it easier to plot significant moderator effects and makes it easy to explain the moderating effect [48].

## 3. Results

### 3.1. Descriptive Analyses

A total of 11,160 students participated in this study, including 5419 (48.6%) boys and 5741 (51.4%) girls. The ages of the students ranged from 10 to 17 years (Mean = 14.34, *SD* = 1.77). Among all participants, 3260(29.2%) reached the cutoff score on the CES-D. The mean depressive symptom, resilience, and prosocial behavior scores were 7.51 (*SD* = 5.74), 38.29 (*SD* = 9.81), and 12.70 (*SD* = 2.18), respectively.

### 3.2. Correlations between Study Variables

Correlations between study variables are indicated in Table 2. The results showed that prosocial behavior was negatively correlated with depression (*r* = −0.40, *p* < 0.001), but positively correlated with resilience (*r* = 0.62, *p* < 0.001). Regarding demographic variables, age was negatively correlated with depression (*r* = −0.08, *p* < 0.001), but positively correlated with both resilience (*r* = 0.15, *p* < 0.001) and prosocial behavior (*r* = 0.09, *p* < 0.001). The education level of the fathers were positively correlated with depression (*r* = 0.17, *p* < 0.001), but negatively correlated with resilience (*r* = −0.25, *p* < 0.001) and prosocial behavior (*r* = −0.16, *p* < 0.001). The education level of the mothers were also positively correlated with depression (*r* = 0.17, *p* < 0.001), but negatively correlated with resilience (*r* = −0.22, *p* < 0.001) and prosocial behavior (*r* = −0.13, *p* < 0.001). There was no significant correlation between gender and depression (*p* > 0.05). Therefore, in the following analyses, age, and education level of the parents were included as covariates [47].

### 3.3. Moderating Analyses

To examine whether resilience moderated the relationship between prosocial behavior and depression, a moderating model was explored. The results showed that the statistical model was significant (*F* (6, 11,153) = 576.54, *p* < 0.001, ΔR^2^ = 0.24). As shown in Table 3, the main predictors were significantly related to depression in these children and adolescents. Specifically, both prosocial behavior (*β* = −0.25, *t* = −21.90, *p* < 0.001) and resilience (*β* = −0.31, *t* = −28.60, *p* < 0.001) negatively predicted depression. Furthermore, resilience significantly moderated the association between prosocial behavior and depression (*β* = −0.08, *t* = −9.89, *p* < 0.001).

**Figure 2:**
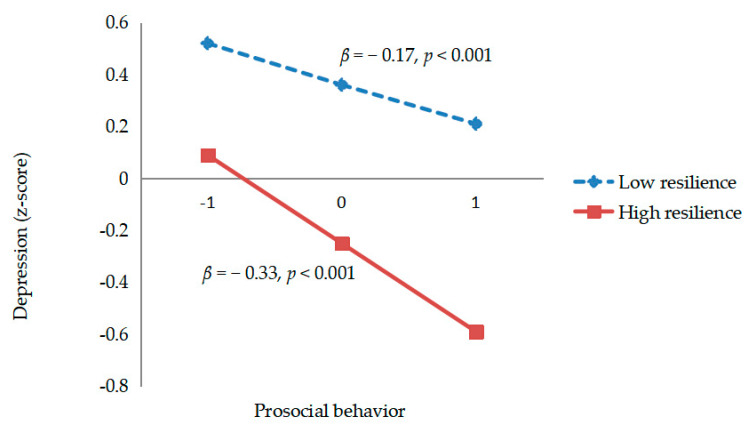
Simple regression lines of prosocial behavior on depression under different levels of resilience. Note: the X-axis represents the level of prosocial behavior; the *Y*-axis represents the z-score of depression; resilience was divided into two groups: low (−1 *SD*) and high (+1 *SD*) levels.

The simple regression lines of prosocial behavior on depression at low (−1 *SD*) and high (+1 *SD*) levels of resilience are shown in Figure 2. The results revealed that the slope was significant for individuals with both low resilience (*β* = − 0.17, *t* = −14.84, *p* < 0.001) and high resilience (*β* = − 0.33, *t* = −20.05, *p* < 0.001). The simple slope value for those with high resilience was higher than that for those with low resilience (− 0.33 vs. − 0.17). That is, resilience may function as a moderator of the relationship between prosocial behavior and depression. Specifically, the association between prosocial behavior and depression would be stronger in individuals with high resilience than in those with low resilience.

## 4. Discussion

To our knowledge, this is the first study to examine the prevalence of depression and to explore the associations between prosocial behavior, depression, and resilience in a large sample of children and adolescents on the Qinghai-Tibet Plateau. The findings provide novel insights for understanding depression among children and adolescents, as well as prosocial behavior and resilience.

Specifically, a considerable number of children and adolescents (29.2%) in the current study met the cutoff for depression. The prevalence of depression in the present study was higher than that in a general population of children and adolescents in China (19.8% to 24.3%) [3,4]. These different findings may be caused by the data collected from different regions. Specifically, the data in this study were collected in the Yushu Prefecture in the northwestern region of China, which has an average altitude of over 4200 m, while the previous research data were mainly from the eastern and southern regions of China, which are at lower altitudes than the Yushu Prefecture. High altitude exposes individuals to chronic hypobaric hypoxia, which may affect emotion regulation and thus further contribute to a higher prevalence of depression [49,50]. Moreover, due to inconvenient transportation, low population density, and less information exchange at high altitudes, it is difficult to access mental health resources [9,12]. In addition, the higher prevalence of depression in the current study may also be explained by the low economic level and poor living conditions in this area [51].

In addition, the present study found that prosocial behavior may play an important role in children’s and adolescents’ depression. Specifically, as hypothesized, prosocial behavior was negatively associated with depression, consistent with previous research indicating that prosocial behavior may protect against depression [22,52]. Previous research has demonstrated that children with high prosocial behavior receive positive feedback and peer acceptance from their peers [53], and likeability and acceptance by peers have been associated with children’s improved confidence and reduced depression [54]. Thus, prosocial behaviors are central to the formation and maintenance of healthy interpersonal relationships [15], which may protect children and adolescents from the risk of maladjustment and further increase positive mood. Moreover, the experience of helping others results in more positive feelings [19]. Thus, children and adolescents with high prosocial behavior exhibit lower levels of depression.

Finally, consistent with our hypothesis, the results showed that the relationship between prosocial behavior and depression was moderated by resilience. Specifically, resilience may enhance the effects of prosocial behavior on depression: in children and adolescents with higher levels of resilience, the relationship between prosocial behavior and depression was significantly stronger than in children and adolescents with lower levels of resilience. This result is in line with previous research indicating that resilience is a protective factor against depression [33,55] and that the moderation of resilience was achieved by enhancing the effects of favorable factors [34].

Previous studies have suggested that individuals with high resilience possess more psychological resources, including flexibility, emotion regulation, coping strategies, and personal characteristics, that increase the availability of social support [25,31]. Moreover, resilient individuals use positive emotions (e.g., humor and positive thinking) to rebound from negative emotional experiences [27]. Thus, individuals with high resilience may have more resourcefulness, positive emotions, and better emotion regulation than those with low resilience, which could be helpful in improving emotional stability and enhancing the positive emotions initiated by prosocial behavior, thereby reducing depression. Furthermore, resilience theory suggests that psychological resources, including positive school experiences and good peer relationships, are protective factors regarding individuals’ mental health [30,56]. Thus, through these resources, resilience may promote high prosocial behavior to enhance positive school experiences and improve harmonious peer relationships that mitigate the levels of depression. In sum, individuals with high resilience may exhibit more prosocial behaviors to cope with difficulties and adapt to the environment, thereby protecting their mental health.

It is important to note that age was negatively correlated with depression but positively correlated with resilience. This may be because older adolescents tended to use a broader range of coping strategies (e.g., problem-solving and emotional regulation) than younger children [57], which was helpful in reducing the level of depression. Meanwhile, the broader range of coping strategies of older adolescents makes them possess more psychological resources to cope with difficulties and show more resilience in adversity [58]. Prosocial behavior and age also showed a positive correlation in the current study. These findings are consistent with some previous studies [59,60], but not with others [61,62]; that is, previous studies have not drawn consistent conclusions about the relationship between age and prosocial behavior. These different results may suggest that the development of prosocial behavior may be complex during childhood and adolescence. More validation is needed in the future. In addition, we also found that the education level of the parents were negatively associated with depression but positively associated with both resilience and prosocial behavior. On one hand, parents with high education level have more ability and resources to protect their children from adversity; thus, their children may be confronted with less threatening stimuli and show lower levels of resilience. On the other hand, parents with higher education level have higher educational expectations and academic performance on their children in China [63], which leads to more academic pressure on children and may increase their depression levels. Meanwhile, children under high academic pressure may regulate their behaviors to pay more attention to their own learning, which will reduce their prosocial behavior [64].

### 4.1. Limitations and Future Directions

Our findings should be considered in light of the following limitations. First, depression was assessed by a self-reported questionnaire rather than a clinical assessment. The use of self-report measures without further clinical observations may only point towards the levels of symptoms and not necessarily imply a clinical diagnosis. Nonetheless, the CES-D-10 is a well-established measure of depression. Future studies should assess depression through clinical observations to replicate and validate our findings. Second, our study was a cross-sectional study and thus could not verify the causal relationships between these variables. Future longitudinal research is needed to better delineate the relationships among prosocial behavior, resilience, and depression. Moreover, we found a negative correlation between prosocial behavior and depression. Another possible explanation of this correlation is that high levels of depression may reduce prosocial behavior, which is related to social withdrawal in depression. The association between prosocial behavior and depression is complex and needs to be further examined. Finally, this study lacks a comparison of depression among children and adolescents between high- and low-altitude areas. Future studies should consider using matched groups to test the effects of high altitude on depression.

### 4.2. Implications

The present study has important practical implications. First, children and adolescents at high altitudes may be at an elevated risk for depression. Hence, we should focus on the mental health of children and adolescents in high-altitude areas and provide more psychological services. Furthermore, families, schools and society should attend more to children and adolescents with fewer prosocial behaviors, as low prosocial behavior may be significantly associated with higher levels of depression. Hence, prevention and intervention strategies should focus on promoting prosocial behavior among children and adolescents, which may help to reduce the levels of depression to some extent. Finally, improving children’s and adolescents’ resilience may also be beneficial by enhancing the association between prosocial behavior and reduced depression, which contributes to children’s and adolescents’ mental health. Resilience can be enhanced with proper interventions. For instance, fostering mutually beneficial relationships is a key strategy to boost resilience [65].

## 5. Conclusions

The prevalence of depression was 29.2% in the current study. Furthermore, higher levels of prosocial behavior were associated with lower levels of depression. Finally, the results suggest that resilience may moderate the association of prosocial behavior with depression, with a stronger moderating effect in individuals with high resilience. Specifically, resilience enhances the protective roles of prosocial behavior against depression. The present study extends our understanding of prosocial behavior, depression, and resilience among children and adolescents on the Qinghai-Tibet Plateau.

## Figures and Tables

**Table 1:** Sociodemographic characteristics of the participants (*N* = 11,160).

Sociodemographic Characteristics	Category	Frequency	%
Gender	Boys	5419	48.6
	Girls	5741	51.4
Age (years)	10–11	702	6.3
	12–13	2780	24.9
	14–15	4423	39.7
	16–17	3255	29.1
^a^ Education level of the fathers	Never went to school	8405	75.3
	Primary school	1131	10.1
	Junior high school	887	7.9
	High school or above	602	5.4
^a^ Education level of the mothers	Never went to school	7758	69.5
	Primary school	998	8.9
	Junior high school	777	7.0
	High school or above	494	4.4

Note: ^a^: there are missing values in sociodemographic characteristics; thus, the sum of the effective percentages is not equal to 100% in these cases.

**Table 2:** Correlations, means, and standard deviations of the study variables (*N* = 11,160).

Variables	1	2	3	4	5	6	7
1. Depression	—	−0.45 ***	−0.40 ***	−0.08 ***	0.02	0.17 ***	0.17 ***
2. Resilience		—	0.62 ***	0.15 ***	0.02	−0.25 ***	−0.22 ***
3. Prosocial behavior			—	0.09 ***	0.01	−0.16 ***	−0.13 ***
4. Age				—	−0.01	−0.15 ***	−0.17 ***
5. Gender					—	−0.01	−0.01
6. Education level of the fathers						—	0.88 ***
7. Education level of the mothers							—
*M*	7.51	38.29	12.70	14.34			
SD	5.74	9.81	2.18	1.77			

Note: gender: 0 = girls, 1 = boys; education level of the fathers and mothers: 0 = never went to school, 1 = primary school, 2 = junior high school, or 3 = high school and above. *** *p* < 0.001. *M* = mean. *SD* = standard deviation.

**Table 3:** Moderating effect of resilience on the associations between prosocial behavior and depression.

Variables	*β*	*SE*	*t*	95% (CI)
Prosocial behavior	−0.25	0.01	−21.90 ***	(−0.27, −0.23)
Resilience	−0.31	0.01	−28.60 ***	(−0.33, −0.29)
Prosocial behavior × Resilience	−0.08	0.01	−9.89 ***	(−0.10, −0.07)
Levels of resilience				
Low (Z = −1.0)	−0.17	0.01	−14.84 ***	(−0.19, −0.14)
High (Z = 1.0)	−0.33	0.02	−20.05 ***	(−0.37, −0.30)

Note: age and education level of the parents were control variables according to our results. *** *p* < 0.001. *SE* = standard error. CI = confidence interval.
