# Erratum: Eli, B., et al. Depression in Children and Adolescents on the Qinghai-Tibet Plateau: Associations with Resilience and Prosocial Behavior. *Int. J. Environ. Res. Public Health* 2021, *18*, 440

**DOI:** 10.3390/ijerph18073707

**Published:** 2021-04-02

**Authors:** Buzohre Eli, Yueyue Zhou, Yiming Liang, Jin Cheng, Jiazhou Wang, Changbing Huang, Xi Xuan, Zhengkui Liu

**Affiliations:** 1 CAS Key Laboratory of Mental Health, Institute of Psychology, Chinese Academy of Sciences, Beijing 100101, China; ail@psych.ac.cn (B.E.); zhouyueyue@psych.ac.cn (Y.Z.); liangym@psych.ac.cn (Y.L.); wangjz@psych.ac.cn (J.W.); 2 Department of Psychology, University of Chinese Academy of Sciences, Beijing 100049, China; 3 School of Psychology, Beijing Sport University, Beijing 100084, China; chengjin2020@163.com; 4 CAS Key Laboratory of Behavioral Science, Institute of Psychology, Chinese Academy of Sciences, Beijing 100101, China; huangcb@psych.ac.cn; 5 Department of Law and Politics, Nankai University Binhai College, Tianjin 300270, China; xuanxi123456@126.com

## Incorrect Text

There was a small mistake in the sixth paragraph of the Discussion: the positions of two words (“positively” and “negatively”) should be exchanged. The correct expression [1] is “In addition, we also found that the education levels of the parents were positively associated with depression but negatively associated with both resilience and prosocial behavior.”

We apologize for any inconvenience caused to the readers by this mistake.
